# Deciphering imprints of impaired memory B-cell maturation in germinal centers of three patients with common variable immunodeficiency

**DOI:** 10.3389/fimmu.2022.959002

**Published:** 2022-10-06

**Authors:** Pauline van Schouwenburg, Susanne Unger, Kathryn J. Payne, Fabian M. P. Kaiser, Ingrid Pico-Knijnenburg, Jens Pfeiffer, Oliver Hausmann, David Friedmann, Michelle Erbel, Maximilian Seidl, David van Zessen, Andrew P. Stubbs, Mirjam van der Burg, Klaus Warnatz

**Affiliations:** ^1^ Laboratory for Pediatric Immunology, Department of Pediatrics, Willem-Alexander Children’s Hospital, Leiden University Medical Center (LUMC), Leiden, Netherlands; ^2^ Department of Immunology, Erasmus University Medical Center, Rotterdam, Netherlands; ^3^ Department of Rheumatology and Clinical Immunology, Medical Center – University of Freiburg, Faculty of Medicine, University of Freiburg, Freiburg, Germany; ^4^ Center for Chronic Immunodeficiency (CCI), Medical Center – University of Freiburg, Faculty of Medicine, University of Freiburg, Freiburg, Germany; ^5^ Faculty of Biology, University of Freiburg, Freiburg, Germany; ^6^ Department of Pediatrics, Erasmus University Medical Center, Rotterdam, Netherlands; ^7^ Department of Otorhinolaryngology- Head and Neck Surgery, University of Freiburg, Freiburg, Germany; ^8^ Löwenpraxis and Klinik St. Anna, Luzern, Switzerland; ^9^ Institute of Surgical Pathology, Department of Pathology, Medical Center - University of Freiburg, Faculty of Medicine, University of Freiburg, Freiburg, Germany; ^10^ Institute of Pathology, Heinrich Heine University and University Hospital of Duesseldorf, Duesseldorf, Germany; ^11^ Clinical Bioinformatics Unit, Department of Pathology, Erasmus University Medical Center, Rotterdam, Netherlands

**Keywords:** germinal center, common variable immunodeficiency (CVID), B-cell differentiation, BCR repertoire analysis, secondary lymphoid organs (SLO), plasma cell differentiation

## Abstract

Common variable immunodeficiency (CVID), characterized by recurrent infections, low serum class-switched immunoglobulin isotypes, and poor antigen-specific antibody responses, comprises a heterogeneous patient population in terms of clinical presentation and underlying etiology. The diagnosis is regularly associated with a severe decrease of germinal center (GC)-derived B-cell populations in peripheral blood. However, data from B-cell differentiation within GC is limited. We present a multiplex approach combining histology, flow cytometry, and B-cell receptor repertoire analysis of sorted GC B-cell populations allowing the modeling of distinct disturbances in GCs of three CVID patients. Our results reflect pathophysiological heterogeneity underlying the reduced circulating pool of post-GC memory B cells and plasmablasts in the three patients. In patient 1, quantitative and qualitative B-cell development in GCs is relatively normal. In patient 2, irregularly shaped GCs are associated with reduced somatic hypermutation (SHM), antigen selection, and class-switching, while in patient 3, high SHM, impaired antigen selection, and class-switching with large single clones imply increased re-cycling of cells within the irregularly shaped GCs. In the lymph nodes of patients 2 and 3, only limited numbers of memory B cells and plasma cells are formed. While reduced numbers of circulating post GC B cells are a general phenomenon in CVID, the integrated approach exemplified distinct defects during GC maturation ranging from near normal morphology and function to severe disturbances with different facets of impaired maturation of memory B cells and/or plasma cells. Integrated dissection of disturbed GC B-cell maturation by histology, flow cytometry, and BCR repertoire analysis contributes to unraveling defects in the essential steps during memory formation.

## Introduction

Common variable immunodeficiency (CVID) is the clinically most prevalent human antibody deficiency disorder (1). Patients are characterized by recurrent infections, low serum IgG and IgA and poor specific antibody responses (2). The disorder comprises a highly heterogeneous patient population in terms of clinical presentation and underlying etiology. Monogenic defects have been identified in only up to 20% of patients (3–6). Clinically, CVID patients can be divided into “infections only” patients (CVIDio) who suffer only from recurrent infections and their sequelae and patients with additional non-infectious complications (CVIDc) such as polyclonal lymphoproliferation, autoimmune cytopenias, enteropathy, hepatopathy, interstitial lung disease, granulomatous disease, and lymphoid malignancy, indicating additional underlying immune dysregulation (7).

CVID has been associated with a cell-intrinsic or -extrinsic block in B-cell development, but the stage at which development is blocked varies (8, 9). Some patients have a block in early B-cell development, but the vast majority of patients have reduced levels of post-germinal center (GC) memory B cells and plasmablasts (PB) in the periphery suggestive of a defect in (post-) GC B-cell differentiation (8–11). Previously, we and others have shown that often CVID patients have not only a quantitative but also a qualitative defect of circulating post-GC cells as memory B cells of the majority of CVID patients have reduced levels of somatic hypermutation (SHM) and less usage of distal constant domains in their *IGHG* and *IGHA* transcripts (12–14). Both quantitative and qualitative defects of post-GC cells can be found as a result of poor GC formation like in ICOS deficiency (15), but it is also seen in CVID patients with preserved GC formation, suggesting that functional defects within the GC reaction can also lead to a CVID phenotype (13, 16, 17). The presence of a GC with defective output provides an opportunity for careful dissection of relevant steps within the GC reaction associated with the failure of B-cell memory formation.

GCs are typically formed after the cognate interaction of T- and B-cells within B-cell follicles of secondary lymphoid organs (SLO) in response to infection or vaccination. Some of the activated B cells class-switch and differentiate into centroblasts (CB) to form the “dark zone” of GCs. After successful proliferation and SHM, B cells differentiate into centrocytes (CC) and migrate along a cytokine gradient to the “light zone” of the GC where B cells with high affinity BCRs are positively selected for survival and further differentiation into PBs and memory B cells by interaction with T follicular helper (Tfh) cells and follicular dendritic cells (FDC). Non-selected B cells may die or return to the dark zone to undergo additional rounds of selection within the GC (18–21).

Despite the extensive literature on the lack of post-GC B cells in peripheral blood of CVID patients, there is only limited data available from the site of their generation: the GCs. Preliminary work showed plasma cell numbers are often reduced in the SLO of CVID patients (17, 22), and in CVIDc patients GCs are irregularly shaped (17). Altered GC function in CVIDc is associated with a T-helper 1 (TH1) shift of Tfh cells and potentially a failure to contain intestinal microbiota (13, 23). In this study we have the unique opportunity to dissect CVID patients’ SLO and investigate in depth the processes impaired in the GC reaction. This was achieved by an integrated multi-technology approach combining histology, flow cytometry, and BCR repertoire analysis in sorted B-cell populations of three patient-derived GCs to further understand both qualitative and quantitative defects in B-cell development of CVID patients.

## Materials and methods

### Patients and controls

Peripheral blood and tissue samples were obtained from CVID patients seen at the immunodeficiency clinic of the Freiburg University Medical Center, the Charité Berlin, or the University Hospital Frankfurt. Patient tonsil (P1) and lymph node (P2 and P3) biopsies were performed for medical reasons because of tonsillitis or to exclude lymphoma in patients with lymphadenopathy and were found to be tumor-free. All patients were adults and fulfilled the criteria for CVID according to the European Society for Immunodeficiencies (ESID) (2). Tonsillar control biopsies were obtained from adult individuals without known immunodeficiency undergoing tonsillectomy (n = 28). The study was approved by local authorities (Freiburg 239/1999 and 121/11) and written informed consent was obtained from all patients and controls.

### Histology

Immunohistochemistry was performed on 2-μm-thick tissue sections with a Dako Autostainer (Dako). For details, see **Supplementary Data**. Antibody stained slides were imaged on an Olympus BX51 microscope (Olympus) equipped with an AxioCam MRc camera (Carl Zeiss Microscopy) and Fiji software was used for image processing (24). Images were adjusted for brightness and contrast, and the background of separated colors was subtracted using the rolling ball algorithm with a radius of 50 pixels.

### Isolation and flow cytometric analysis of peripheral blood mononuclear cells and mononuclear cells from secondary lymphoid organs

PBMCs were isolated by Ficoll density centrifugation (Pancoll, Pan-Biotech). Mononuclear cells from SLO were isolated by mechanical disruption as previously described (25). PBMC, lymph node-derived, and tonsillar mononuclear cells were labeled with the respective antibodies for detection of the indicated cell surface molecules (**Supplementary Data**). Data were acquired on a Gallios^®^ flow cytometer (Beckman Coulter, Brea, CA, USA) or LSR II^®^ flow-cytometer (BD Biosciences, Franklin Lakes, NJ, USA) and analyzed using the FlowJo^®^ software (version 7.6.5 or 10) (Treestar, Ashland, OR, USA).

### Classification of B-cell populations by flow cytometry

For circulating B cells, transitional B cells were defined as CD19^pos^CD21^pos^CD38^hi^IgD^pos^IgM^hi^CD27^neg^, naïve B cells as CD19^pos^CD21^pos^CD38^pos^IgD^pos^CD27^neg^, natural effector (IgM memory B cells) as CD19^pos^CD21^pos^CD38^hi^IgD^pos^CD27^pos^, class-switched memory B cells as CD19^pos^CD21^pos^CD38^pos^IgD^neg^CD27^pos^, IgG^pos^ memory B cells as CD19^pos^CD21^pos^CD38^pos^IgD^neg^CD27^pos^IgG^pos^, IgA^pos^ memory B cells as CD19^pos^CD21^pos^CD38^pos^IgD^neg^CD27^pos^IgA^pos^, IgG^pos^ atypical memory B cells as CD19^pos^CD21^pos^CD38^pos^IgD^neg^CD27^neg^IgG^pos^, IgA^pos^ atypical memory B cells as CD19^pos^CD21^pos^CD38^pos^IgD^neg^CD27^neg^IgA^pos^, PB as CD19^pos^CD21^pos^CD38^hi^IgD^neg^CD27^hi^, CD21lowCD38low B cells as CD19^pos^CD21^low^CD38^low^

For secondary lymphoid organs, Naïve CD19^pos^CD21^pos^CD38^pos^IgD^pos^CD27^neg^, GC B cells as CD19^pos^CD21^pos^CD38^mid^IgD^neg^, CC GC B cells as CD19^pos^CD21^pos^CD38^mid^IgD^neg^CXCR4^neg^, CB GC B cells as CD19^pos^CD21^pos^CD38^mid^IgD^neg^CXCR4^pos^, IgG^pos^ GC B cells as CD19^pos^CD21^pos^CD38^mid^IgD^neg^IgG^pos^, IgA^pos^ GC B cells as CD19^pos^CD21^pos^CD38^mid^IgD^neg^IgA^pos^, memory of B cells as CD19^pos^CD21^pos^CD38^neg^IgD^neg^, IgG^pos^ memory B cells CD19^pos^CD21^pos^CD38^neg^IgD^neg^IgG^pos^, IgA^pos^ memory B cells CD19^pos^CD21^pos^CD38^neg^IgD^neg^IgA^pos^, IgM^pos^ memory B cells CD19^pos^CD21^pos^CD38^neg^IgD^neg^IgM^pos^, plasmablasts B cells as CD19^pos^CD21^pos^CD38^high^IgD^neg^

### Repertoire sequencing

RNA was isolated from PBMC and SLO sorted populations using RNeasy Plus Kits (Qiagen) and transcribed into cDNA using random hexamer primers and Superscript III^®^ (both Invitrogen). IGH transcripts of the SLO samples were amplified from 5 µl cDNA per reaction in a multiplex PCR using the forward VH1-6 FR1 (BIOMED-2) primers and the CgCH1 (26), the IGHA, or the IGHM reverse primer (sequence available upon request) (27). These PCR products were purified and sequenced using Roche 454 sequencing as previously described (see also **Supplementary Data**) **(** 28). IGH transcripts of the PBMC samples were amplified using the forward VH1-6 FR1 (BIOMED-2) primers and costum IGHG, IGHA, and IGHM reverse primers (for template-specific sequences see **Supplementary Table 1**) (25). PCR products were purified and sequenced using 2x300 Illumina sequencing (Illumina). The filtering and removal of sequencing errors is described in the **Supplementary Data** (**Supplementary Figure 1** and **Supplementary Table 2** **)**.

## Results

In this study we combined the histology, flow cytometry, and BCR repertoire of sorted GC B-cell populations to dissect the heterogeneous dysregulations in B-cell maturation and differentiation in the GC of three CVID patients (P1-3) with reduced circulating switched memory B cells (**Tables 1**, **2**). P1 suffered from CVIDio, while both P2 and P3 belonged to the CVIDc subgroup.

**Table 1:** Frequencies of B-cell subsets in blood.

B-cell subsets in blood (all populations calculated as % of total B cells)	P1	P2	P3	Internal laboratory reference range
Transitional B cells	10.1	3.2	11.9	0.5–4.4
Naive B cells	70.0	74.9	57.4	39.5–76.3
Natural effector (IgM memory B cells)	9.5	6.1	5.4	4.4–26.2
Class-switched mem B cells	1.95	0.2	0.7	5.7–24.0
IgG^pos^ mem B cells	0.0	0.1	0.0	2.5–20.3
IgA^pos^ mem B cells	0.0	0.0	0.0	2.8–10.9
IgG^pos^ atypical mem B cells	0.7	0.1	0.0	0.8–7.2
IgA^pos^ atypical mem B cells	0.0	0.0	0.0	0.4–2.2
Plasmablasts	0.33	0.0	0.1	0.2–3.4
CD21^low^CD38^low^ B cells	2.0	15.5	22.6	1.3–7.3

Blue and red color highlight: patient values below and above the 10 and 90 percentiles of HDs, respectively. mem, memory. Population definitions refer to Classification of B cell populations by flowcytometry.

**Table 2:** Frequencies of B-cell subsets in SLO.

B-cell subsets in SLO	P1	P2	P3	Mean and 10–90 percentile in HD tonsils *
% Naive of total B cells	65.6	56.6	41.1	42.5 (29.2–57.5)
% GC of total B cells	7.9	14.5	28.0	20.6 (11.2–32.0)
% Centroblasts of GC	77.2	71.2	64.7	63.8 (54.4–76.2)
% Centrocyte of GC	19.5	24.9	30.2	31.3 (20.3–38.5)
% IgG^pos^ of GC	54.0	13.9	11.6	37.5 (16.1–57.9)
% IgA^pos^ of GC	19.8	0.1	0.0	26.3 (12.4–51.4)
% mem of total B cells	14.3	5.5	0.9	14.8 (6.3–24.0)
% IgG^pos^ of mem	49.0	8.1	1.5	40.4 (18.9–57.0)
% IgA^pos^ of mem	17.5	0.0	0.0	25.3 (16.5–38.5)
% IgM^pos^ of mem	1.8	56.6	71.6	4.9 (1.75–10.7)
% Plasmablasts of total B cells	1.3	0.4	0.3	1.1 (0.4–1.9)

Blue and red color highlight: patient values below and above the 10 and 90 percentiles of HDs, respectively. GC, germinal center; mem, memory. Population definitions refer to Classification of B cell populations by flowcytometry.

### Differences in germinal center shape and cellular differentiation in two CVID patients

**Figure 1:**
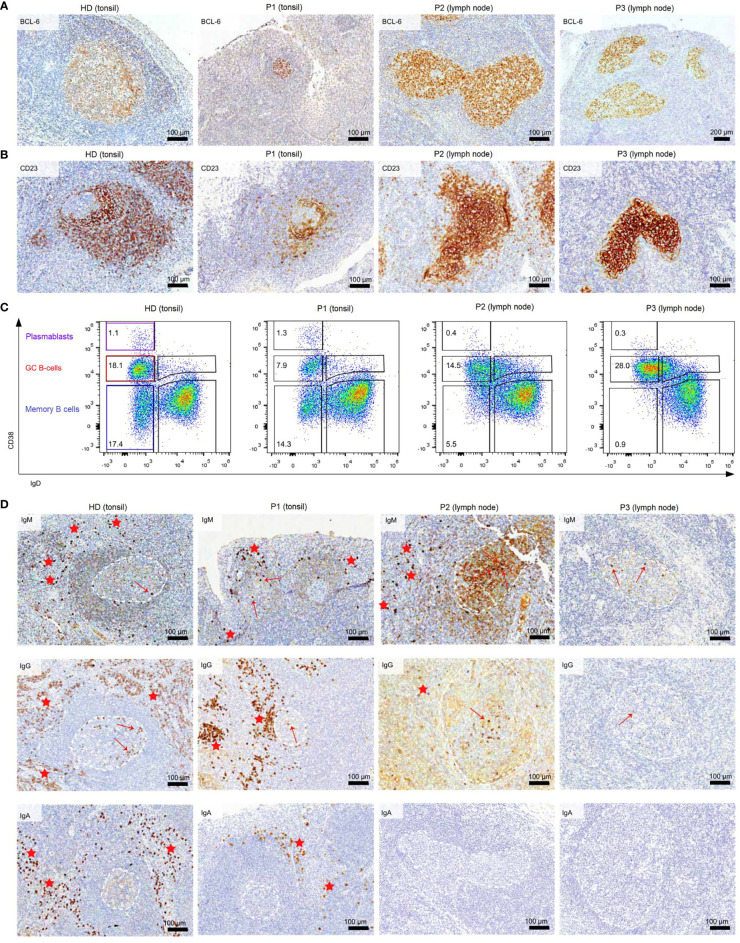
Dysmorphic GCs and impaired B-cell differentiation in CVID patients **(A)** BCL6 staining of tonsils and lymph nodes of HDs and CVID patients. **(B)** CD23 staining of tonsils and lymph nodes to highlight follicular dendritic cells present in the GC light zone as an indicator of GC polarization. **(C)** Frequency of GC, memory B cells, and PB in total B cells of secondary lymphoid organs. **(D)** Clusters of extrafollicular IgM, IgG, and IgA plasma cells in secondary lymphoid organs are highlighted by red stars, intrafollicular plasma cells by a red arrow. GCs are marked with dashed white lines. The light brown staining in the GC of P2 is due to background staining and does not represent plasma cells.

Histology of the tonsil of P1 showed smaller GCs and a reduced density of B-cell follicles compared to GCs of healthy donors (HDs) with recurrent tonsillitis. The GCs were normal in shape (**Figures 1A, B** **)** and the CB : CC ratio was at the higher end of the normal range (**Supplementary Figures S2A, B**). Histological examination of the lymph nodes of P2 and P3 revealed large irregular-shaped GCs, as previously found in other CVIDc patients (**Figure 1A**) (17). In both patients, staining for follicular dendritic cells (CD23) could not detect the polarization of the GCs (**Figure 1B**). Still, a normal CB : CC ratio was detected by flow cytometry (**Supplementary Figures S2A, B**).

Flow cytometry revealed normal numbers of memory B cells in the SLO of P1 but reduced levels in peripheral blood (**Figure 1C** and **Table 1**). In contrast, switched memory B cells of P2 and P3 were reduced in SLO (**Table 2**; **Figure 1C**) and nearly absent from blood (**Table 1**).

The frequency of total plasmablasts was normal in the SLO of P1, but low in P2 and P3 (**Figure 1C**; **Table 2**). The isotype and location of plasma cells strongly differed between the three patients. In P1 all three isotypes were detectable and the distribution of the intra- *vs*. extrafollicular location of all three isotypes was comparable to the findings in HDs (**Figure 1D**). In P2, IgM plasma cells were readily detectable outside the follicle and greatly increased within the follicle. IgG plasma cells, however, were strongly reduced and only located inside the follicles, and IgA plasma cells were undetectable. In P3, only a few intrafollicular IgM plasma cells and nearly no IgG or IgA plasma cells were detectable (**Figure 1D**).

In summary, despite small GCs in P1, class-switch recombination and memory B-cell differentiation appeared rather normal, but low numbers of circulating post-GC B cells suggest defective migration to or survival of memory cells in the periphery. In contrast, both CVIDc patients (P2 and P3) had disturbed class-switching, large and dysmorphic GCs, and impaired GC output, comparable to the majority of patients in our previous study (17). In P2, there were still a strong intrafollicular IgM and detectable IgG plasma cell differentiation and even some extrafollicular IgM plasma cells detectable, whereas in P3, the response was reduced to a few intrafollicular IgM plasma cells.

### Analysis of BCR specification

To get additional insight into BCR specification (SHM, antigen selection, and class switch recombination), we amplified and sequenced the BCR encoding heavy chain gene of sorted populations from SLO (naïve B-cells, CB, CC, mem B cells and PB; refer to Section 2.4 for definition of populations) and blood (naïve B cells, natural effector (NE), memory B cells, and PB). Unfortunately, due to severely reduced B-cell counts in blood, not enough B cells were available from P3 for repertoire analysis, and no data is available for SLO PB of one of the HDs and naïve cells of the SLO of P3. In P3, IgA^pos^ cells were absent and, consequently, *IGHA* transcripts could not be amplified for any of the populations.

BCR sequences were filtered to only include one sequence per clone to preclude skewing by large clones (**Supplementary Figure S1** and **Material and Methods**). Filtering resulted in 0–22591 sequences per class (*IGHM*, *IGHG*, *IGHA*) per population. Populations in which filtering resulted in <45 sequences were excluded from further analysis. This applied to *IGHA* transcripts of P2 (all populations except blood PB) in line with low numbers of IgA^pos^ cells. The few *IGHA* transcripts that were found in P2 were highly clonally related, a finding not observed for other isotypes of P2 (data not shown).

Analysis of parameters for SHM, antigen selection subclass distribution, and clonal relation showed little variation between the three HDs. In contrast, all three CVID patients showed distinct alterations reflecting the heterogeneous nature of CVID. Therefore, results will be described separately for each patient in the following sections.

### Normal BCR repertoire specification in the GC of P1

**Figure 2:**
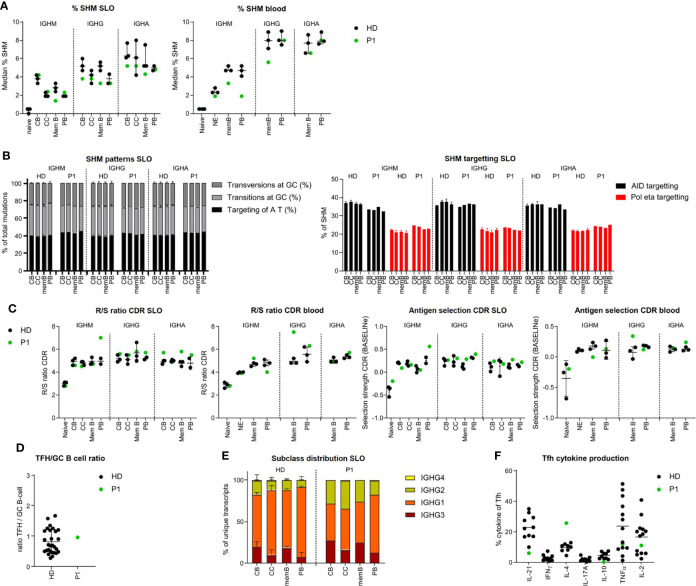
BCR repertoire analysis shows minor differences in BCR specification in the tonsil and blood of P1. **(A)** Median percentage of SHM in *BCR* transcripts of sorted B-cell populations. **(B)** SHM patterns and targeting in sorted B-cell populations. **(C)** Evaluation of antigen selection in sorted B-cell populations. **(D)** Tfh/B-cell ratio in the tonsil. **(E)** Subclass distribution in *IGHG* transcripts of sorted tonsil populations. **(F)** Cytokine production of Tfh cells.

In P1, only minor differences in the BCR repertoire were found compared to healthy controls. SHM levels were mildly reduced in the *IGHG* of CB, *IGHM*, and *IGHG* transcripts of memory B cells (SLO and blood) and the *IGHM* transcripts of circulating PB (**Figure 2A**). This was not the result of impaired processing of AID-induced U lesions as the patterns of SHM in P1 were normal (**Figure 2B** and **Supplementary Figure, S3A**). Parameters for antigen selection did not show clear differences (**Figure 2C** and **Supplementary Figure S3B**) except an increased antigen selection in the *IGHG* transcripts of circulating memory B cells and the *IGHM* transcripts of SLO PB. In addition, both the GC Tfh/B-cell ratio and the IgG subclass distribution were normal (**Figures 2D, E** and **Supplementary Figure S3C**). Analysis of the cytokine production of Tfh cells revealed an increased percentage of IL-4^pos^ and a reduced percentage of IL21^pos^ and low percentage of IL-10^pos^ Tfh cells (**Figure 2F**).

Together, these data suggest that in P1, both quantitative and qualitative GC-dependent B-cell development were relatively normal, corroborating the suggested post-GC defect in this patient.

### Impaired repertoire diversification, antigen selection, and skewed subclass distribution in P2

**Figure 3:**
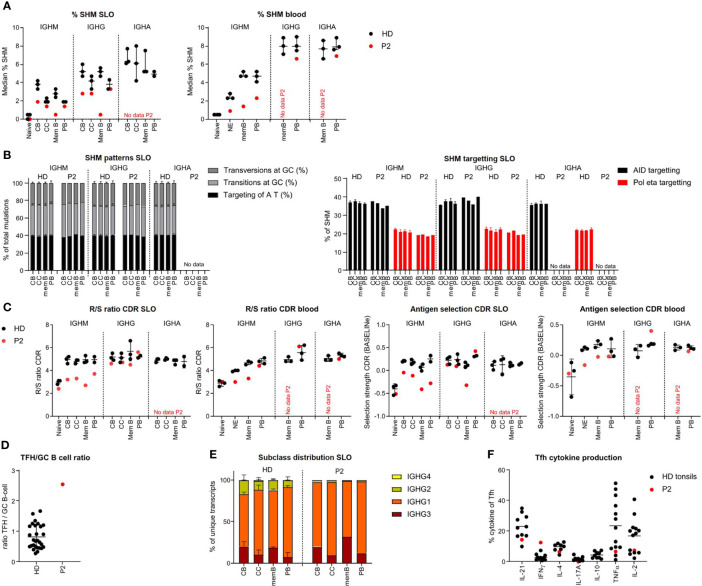
BCR repertoire analysis reveals reduced SHM, impaired antigen selection and altered subclass distribution in P2. **(A)** Median percentage of SHM in *BCR* transcripts of sorted B-cell populations. **(B)** SHM patterns and targeting in sorted B-cell populations. **(C)** Evaluation of antigen selection in sorted B-cell populations. **(D)** Tfh cell/B-cell ratio in the lymph node. **(E)** Subclass distribution in *IGHG* transcripts of sorted lymph node populations. **(F)** Cytokine production of Tfh cells.

In the lymph node of P2, SHM levels were reduced in *IGHM* and *IGHG* transcripts of CB and nearly absent in memory B-cells but within the normal range for CC and PB. In peripheral blood, *IGHM* transcripts of all memory/PB populations showed reduced SHM levels, while SHM levels in *IGHG* and *IGHA* transcripts of the few blood-derived PB were within the normal range (**Figure 3A**). SHM patterns and targeting were normal in all populations (**Figure 3B** and **Supplementary Figure S3A**).

In HDs, SHM was lower in the *IGHM* and *IGHG* transcripts of CC as compared to CB (unpaired t-test: p = 0.026 and p = 0.0094, respectively), most likely reflecting antigen selection. This difference was limited (*IGHM)* or absent (*IGHG*) in P2. Accordingly, parameters for antigen selection were reduced in the *IGHM* transcripts of all GC and memory/PB populations (**Figure 3C** and **Supplementary Figure S3B**), whereas they seemed to suggest normal selection in *IGHG* transcripts of all, but memory, populations. Similarly, antigen selection was reduced in circulating NE and IgM memory B cells but seemed normal in the few blood-derived PB. Antigen selection in GC of P2 may be disturbed by an increased GC Tfh/B cell ratio, as a balanced Tfh/B-cell ratio is essential for competition between B cells for Tfh cell help during antigen selection (**Figure 3D**).

Analysis of subclass distribution of *IGHG* transcripts of GC B-cell populations revealed less usage of the more distal constant domains (**Figure 3E**). The altered subclass distribution in P2 was associated with a skewing of Tfh cells towards a TH1 phenotype (**Figure 3F**) as previously reported (25).

Together, this indicates that P2 had an impaired induction of SHM and a defect in antigen selection possibly due to a decreased Tfh/GC B-cell ratio and a reduced switching to more distal constant regions associated with a TH1-skewed Tfh cytokine production. This is associated with a decreased exit of IgM^pos^ PB from GCs.

### Impaired antigen selection despite increased SHM in CC of P3

**Figure 4:**
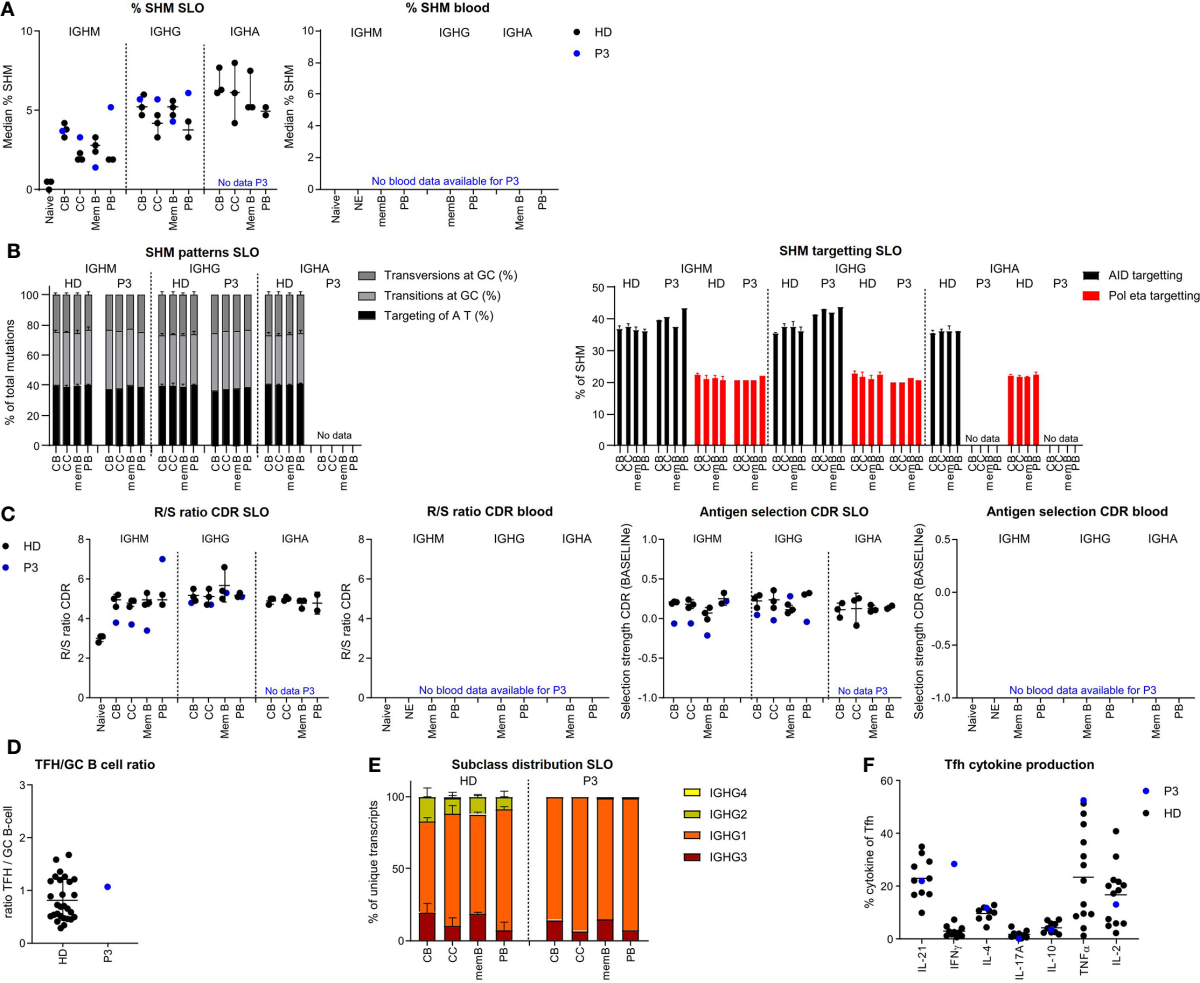
BCR repertoire analysis reveals increased SHM, impaired antigen selection, and altered subclass distribution in P3. **(A)** Median percentage of SHM in *BCR* transcripts of sorted B-cell populations. **(B)** SHM patterns and targeting in sorted B-cell populations. **(C)** Evaluation of antigen selection in sorted B-cell populations. **(D)** Tfh/B-cell ratio in the lymph node. **(E)** Subclass distribution in *IGHG* transcripts of sorted lymph node populations. **(F)** Cytokine production of Tfh cells.

In P3 we found a slight reduction of SHM levels for *IGHM* and *IGHG* transcripts of memory B cells, while SHM levels were normal for CB and even increased in the *IGHM* and *IGHG* transcripts of the CC and PB of the SLO (**Figure 4A**). SHM patterns were normal in all populations (**Figure 4B**, **Supplementary Figure S3A**). Parameters for antigen selection were reduced in the *IGHM* transcripts of CB, CC, and memory B cells. In line with impaired antigen selection, the difference in SHM between CB and CC was very limited (*IGHM*) or absent (*IGHG*) (**Figure 4C** and **Supplementary Figure S3B**). Interestingly, antigen selection was high/normal in the *IGHM* transcripts of PB. Similar to our findings in P2, we found a rather normal selection of *IGHG* transcripts. The Tfh/GC B-cell ratio in P3 was normal excluding a disbalance between Tfh and GC B-cells as a possible cause of impaired selection (**Figure 4D**). *IGHG* transcript analysis revealed a reduced usage of more distal constant domains (**Figure 4E**). This was associated with an increase in interferon gamma (IFN-γ)pos Tfh cell and high tumour necrosis factor alpha (TNF-α)pos Tfh cell percentage potentially altering the GC response (**Figure 4F**).

The normal to high mutation rate seen in CC and CB was not associated with normal antigen selection strength. Similar to P2, this takes place within a TH1-skewed environment, but unlike P2 in the context of a normal Tfh/GC B-cell ratio. A possible explanation of the high SHM levels in P2 may be increased re-cycling between the CB and CC stages.

### Clonal expansion and increased relation of GC populations in P3

**Figure 5:**
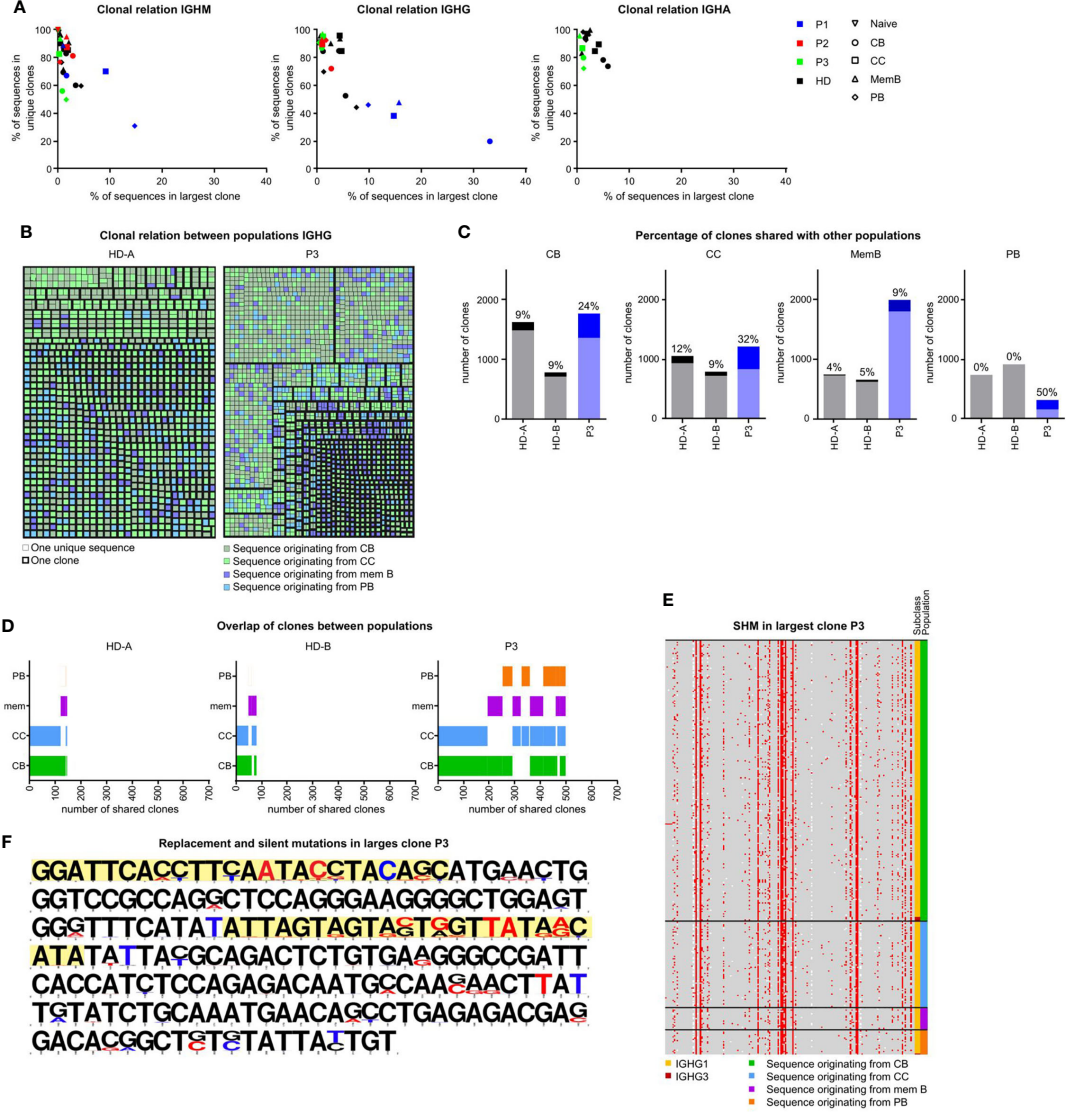
Clonal relation between SLO B-cell populations in P3. **(A)** Clonality of *IGHM*, *IGHG* and *IGHA* transcripts in sorted GC B-cell populations. Samples with little clonality are top left, samples containing one very large clone are bottom left. Samples containing many small clones are bottom right. **(B)** Clonal relation between *IGHG* sequences of HDA and P3. Each colored square represents a single unique sequence, with the color representing the population the sequence originates from. The bold border visualizes which sequences are clonally related. **(C)** The number of clones analyzed in each individual. Clones containing sequences from at least one other population are a darker shade and the percentage is listed on top of each bar. **(D)** Visualization of clones containing sequences in multiple populations. The overlap in bars visualizes the populations with shared clones. **(E, F)** Alignment of all sequences of the largest *IGHG* clone in P3. **(E)** Locations of SHM are indicated in red. The original population and subclass are indicated. **(F)** The original sequence is in black and mutations are indicated in red (replacement mutations) or blue (silent mutations). The CDR regions are highlighted in yellow.

In order to investigate clonal expansion and the clonal relationship between different B-cell populations of the SLO of our patients, we re-analyzed all SLO BCR sequences using alternative filtering. We only included unique sequences (to remove any amplification bias) and vastly reduced sequencing errors (see material and methods for details and **Supplementary Figure S1B**). It should be noted that the analysis of clonal expansion was limited by the number of cells that could be analyzed. The analysis revealed increased clonality in the *IGHM* sequences of the CC and PB and the *IGHG* transcripts of all populations in P3, but not the other patients (**Figure 5A**, data not shown). Additional analysis of the *IGHG* transcripts of P3 confirmed the presence of large clones which primarily consisted of transcripts originating from CB and CC (**Figure 5B**).

To further quantify our findings, we determined the percentage of clones in each population that also contained sequences originating from a different population (**Figure 5C**). Interestingly, the clonal overlap between populations was increased for all populations of P3. Most remarkably, 50% of PB clones of P3 were shared with at least one other population as opposed to 0% in both HDs. In both HDs there was mainly a clonal overlap between the CB and CC population, while in P3, the clonal overlap between all populations was broader (**Figure 5D**).

Increased recycling of cells would result in many shared mutations within clones, whereas impaired selection would result in many variations in mutations within one clone. As shown in **Figures 5E, F**, the largest *IGHG* clone of P3 contained many shared, but also unique mutations. This was in line with a multiple step formation of this clone which may be the result of the recycling of cells between the CB and CC stages. In conclusion, P3 had increased SHM in the CC and PB populations possibly explained by a combination of impaired antigen selection and increased recycling of cells between the CB and CC stages and the exclusive intrafollicular location of PB.

## Discussion

**Figure 6:**
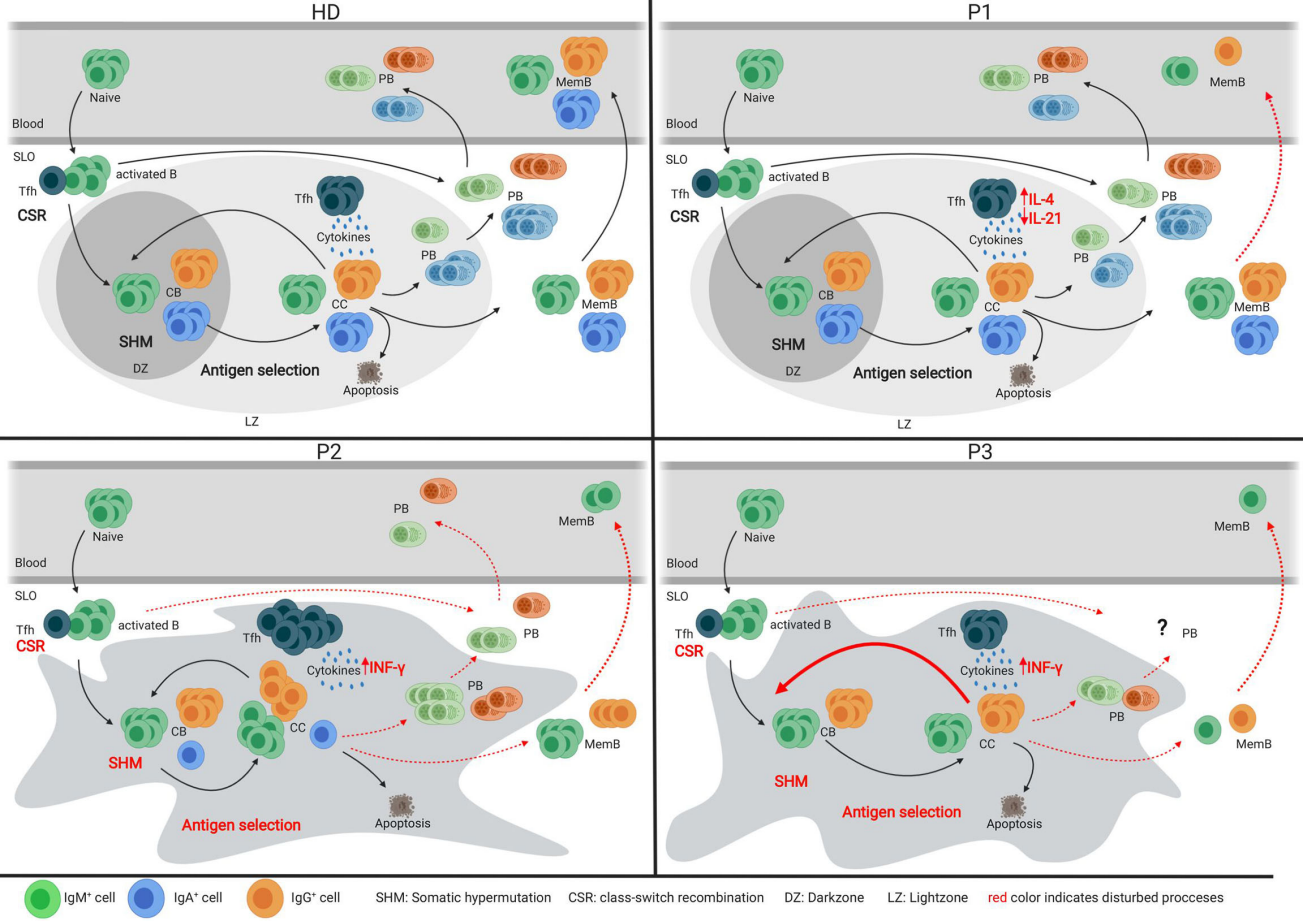
Models of the germinal center of HDs and three CVID patients. Models depicting the germinal center response in HDs and three individual CVID patients. Red font indicates impaired processes in the patient’s GC response.

With the unique opportunity to assess both SLO and blood samples of one CVIDio and two CVIDc patients, we were able to combine the histological, flow cytometric, and BCR repertoire analysis of sorted B-cell populations to identify some differences in the GC-based pathogenesis of the common severe reduction of circulating memory B cells (**Figure 6**).

Analysis of the tonsil of the CVIDio patient P1 showed only very minor abnormalities in GC formation, BCR specification, and B-cell differentiation. This implies (close to) normal B-cell development within the GC and, based on histology, even normal extrafollicular plasma cell responses. However, reduced numbers of circulating class-switched memory B cells indicate that despite normal generation in the GC these cells are unable to exit the GC and/or survive in the periphery (**Figure 6**). It remains speculative how much the reduced size of GC (29) and the lack of long lived humoral response (30) is related to the reduced IL-21 and/or IL-10 production itself or to the altered ratio of IL-4/IL-21 and IL-10 production of Tfh cells in this patient.

In the lymph node of P2, a CVIDc patient, GCs were present, but large, irregular in shape, and without normal polarization (**Figure 6**). The differentiation and ratio of CB and CC were normal, but a severely compromised GC function became evident by low numbers of class-switched and absent IgA memory B cells, a heavily biased IgG sub-class distribution, low SHM rate, and impaired antigen selection. The residual capacity for CSR in P2 was demonstrated by a few IgG-positive cells, *in vitro* CSR to IgE [patient P3 in (22)], and especially IgG production of B cells transferred in the presence of CD4 T cells into a RAG-2/common-γ-chain-deficient mouse (22). Despite the detectable capacity for CSR, IgM^pos^ cells were overrepresented among memory and plasma cell responses in the SLO of P2. Histology showed not only increased numbers of intrafollicular IgM plasma cells but also some in the extrafollicular zone, while the few IgG plasma cells were restricted to the intrafollicular location. Previously, we had shown that plasma cell differentiation did not reach the final Syndecan^hi^Blimp1^low^ stage in this patient [P3 in (22)]. The impaired humoral memory formation is associated with an increased Tfh/GC B-cell ratio and percentage of IFN-γ producing Tfh cells. Both features have been observed in HIV patients and simian immunodeficiency virus models, and were shown to interfere with effective positive selection and development of high-quality HIV broadly neutralizing antibodies and their class-switch to IgG or IgA (31–33). Acute Malaria infection also promotes a TH1 polarization of circulating Tfh cells, which provide suboptimal help to B cells and do not correlate with a good malaria-specific antibody response (34). The observed retention of plasma cells within the follicle has been associated with T-bet expression in c-myb haploinsufficient B cells (35), suggesting a possible role for TH1 driven responses in this phenomenon. Interestingly, there was a strong discrepancy between the very low SHM rate in memory B cells despite the normal SHM pattern and the rather normal SHM rate in plasmablasts. Despite the presence of IgG mem B cells, plasmablasts, and plasma cells in the SLO, none of those post-GC cells made it into circulation, suggesting an additional block at this stage.

In contrast to P2, the irregularly shaped and non-polarized GCs in P3 contained very few class-switched memory B cells. The somatic mutation rate was normal in CB and even increased in CC, but similar to P2, not associated with a normal antigen selection of unswitched memory cells, despite a normal Tfh/GC B-cell ratio. Interestingly, this was not the case for the few IgM plasma cells and the IgG memory B cells in the SLO of P3. Plasma cells are nearly absent and confined to the GC area. There was no detectable extrafollicular plasma cell response. Although of nearly normal quality, only very few IgG memory B cells are formed in the SLO, which become undetectable in the periphery.

Genomic analysis of P1 and P2 did not reveal mutations in known genes associated with primary immunodeficiencies, but P3 carries a heterozygous loss of function mutation in NFKB1 associated with the clinical and immunological phenotype previously described for NFKB1 haploinsufficiency (4, 36–38). *NFKB1* encodes the protein p105, which is processed during the activation of the canonical NF-κB signaling pathway into the active p50. The canonical NF-κB pathway plays a crucial role in several steps of the GC response, including B cell activation, proliferation, and survival (39); GC maintenance and selection (40); and plasma cell differentiation (41). While GC formation and induction of SHM takes place despite the disturbed NF-κB signaling in P3, there were defects in antigen selection possibly associated with increased CB-CC cycling and severely impaired PB differentiation. This phenotype is compatible with the reported critical role of NF-κB signaling downstream of CD40 in GC B cells, which modulates the transcription factors c-Myc and Foxo1 that underlie selection (40) and promotion of plasmablast development due to B-cell intrinsic and extrinsic functions (41–44). It remains to be seen whether the accumulation of non-selective somatic mutations observed in this patient is a general phenomenon in NFKB1 haploinsufficient patients and whether this contributes to an increased risk of GC B-cell derived lymphomas as has been observed (45) among 157 patients with heterozygous NKFB1 mutations (46–49).

Although the three examples of impaired GC function in CVID suggest different underlying pathogenesis, there were also some remarkable overlapping findings. Both P2 and P3 showed reduced antigen selection in *IGHM* transcripts in various GC populations while antigen selection was normal in the *IGHG* transcripts of the same populations. It is therefore tempting to speculate that the few class-switched activated B cells entering the GC may undergo a more vigorous process of antigen selection than non-switched cells (21). We have previously studied *IGHG* and *IGHA* transcripts in the blood as a read-out for the GC response in CVID patients and found very limited signs of impaired antigen selection in CVID patients (12). With the current knowledge, we suggest to include the *IGHM* transcripts of the post-GC cells of these patients in further studies as this might provide a better read-out for impaired antigen selection.

A single cell would be preferable to bulk BCR repertoire analysis given the large differences, especially in SHM levels, between CB, CC, memory B cells, and PB of P2 and P3. Interestingly, in both patients, cells that acquired more SHM (and presumably carried a BCR with a higher affinity) were found among PB and cells with low SHM rate among memory B-cells. These findings fit the model in which high-affinity GC B-cells are selected to become PB, medium-affinity cells recycle in the GC, and low-affinity B cells differentiate into memory B cells (20). This situation can be masked by a strong extrafollicular PB response, which is characterized by PB with low SHM (50). The latter could explain the higher SHM levels in memory B cells compared to PB as seen in HD SLO and would also explain the stronger clonal relationship of PB and the GC-derived B-cell populations in CVID patients with poor extrafollicular response.

While the low numbers of patient samples and the limited material available for research clearly presents a limitation in this study, the integrated analysis of B-cell differentiation at the site of differentiation of three CVID patients demonstrates that patients with a comparable absence of post-GC B cells in peripheral blood can have very different errors of B-cell maturation in the GC response. Including more patients with genetically defined PID will not only provide insight into the molecular pathways deregulated in affected patients and a diagnostic reference map for genetically undefined PIDs but will be of high relevance for our comprehension of GC-derived autoimmune disease and lymphoma development.

## Data availability statement

The data presented in the study are deposited in the GEO repository, accession number GSE213361 https://www.ncbi.nlm.nih.gov/geo/query/acc.cgi?acc=GSE213361.

## Ethics statement

This study was reviewed and approved by Freiburg 239/1999 and Freiburg 121/11. The patients/participants provided their written informed consent to participate in this study.

## Author contributions

PvS performed repertoire analysis, developed models, and wrote the manuscript. SU coordinated the study; planned, performed, and analyzed flowcytometric experiments; co-analyzed the repertoire sequences; and wrote the manuscript. KP edited the final manuscript. MvdB planned and supervised repertoire sequencing and edited the final manuscript. FK and OH, provided patient information. DF performed the sample preparation and flowcytometric experiments. ME and MS provided histology. DvZ and AS supported bioinformatic repertoire analysis. KW had initiated the project, supervised experiments, co-wrote and edited the final manuscript. All authors contributed to the article and approved the submitted version.

## Funding

This study was supported by the Bundesministerium für Bildung und Forschung (BMBF), grant number BMBF 01EO1303 and GAIN 01GM1910A to KW, by the Stichting Sophia Kinderziekenhuis Fonds (grant no. S15-07 Genes and Immunity in SCID) (FK and MB.), and by the Dutch Organization for Scientific Research (NWO/ZonMw veni grant 91616058 to PS) and an EFIS-IL-Short-Term Fellowship to PS.

## Acknowledgments

The authors would like to thank Marion Klima and Monika Erler for patient care, and the patients for their willingness to participate in this study.

## Conflict of interest

The authors declare that the research was conducted in the absence of any commercial or financial relationships that could be construed as a potential conflict of interest.

## Publisher’s note

All claims expressed in this article are solely those of the authors and do not necessarily represent those of their affiliated organizations, or those of the publisher, the editors and the reviewers. Any product that may be evaluated in this article, or claim that may be made by its manufacturer, is not guaranteed or endorsed by the publisher.
