# Supplementary figures and images for: Deciphering imprints of impaired memory B-cell maturation in germinal centers of three patients with common variable immunodeficiency
Source: Front Immunol. 2022 Oct 6;13:959002. doi: 10.3389/fimmu.2022.959002 (PMC9582261; doi:10.3389/fimmu.2022.959002)

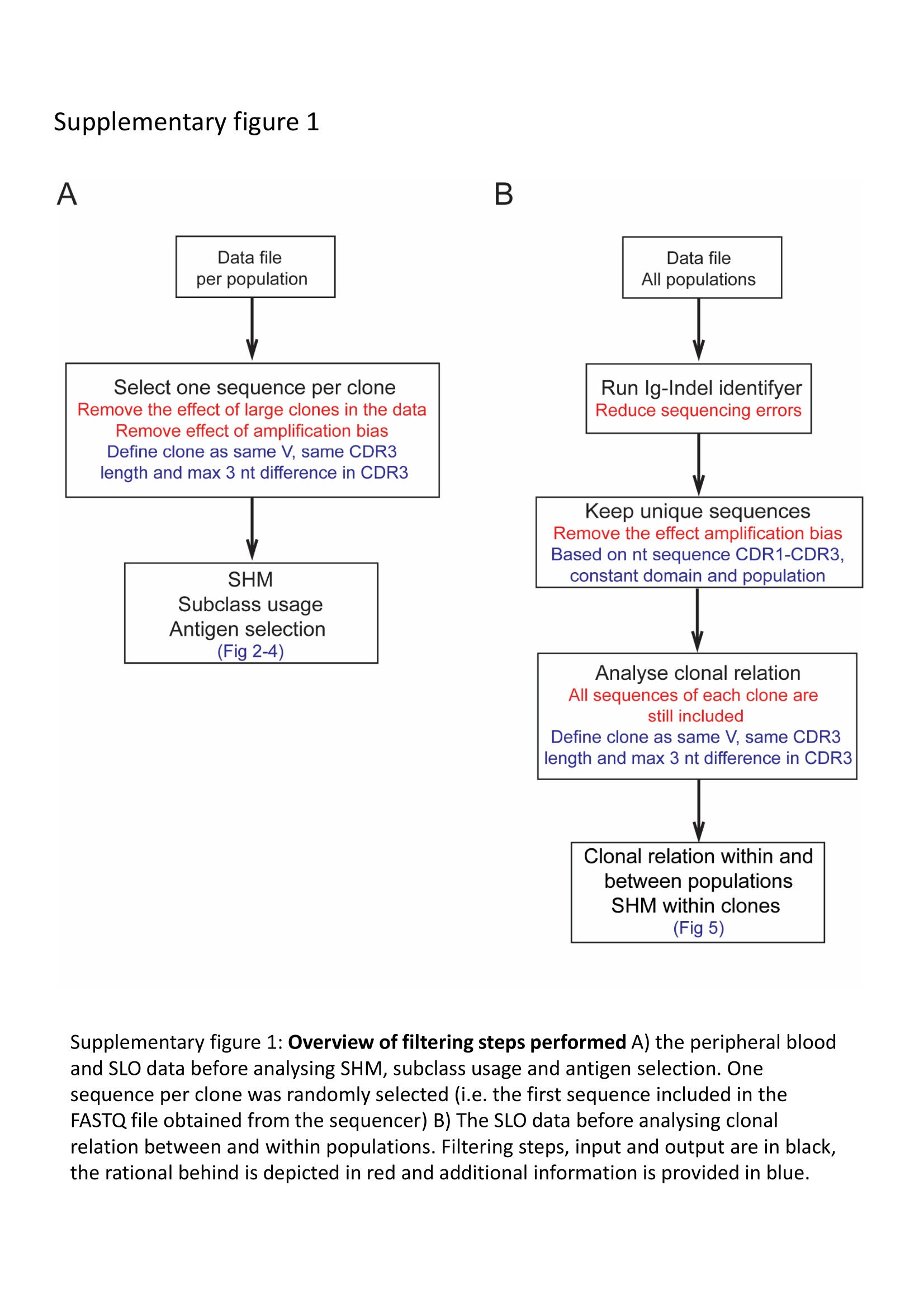

Supplement: Supplementary file 1 [file Image_1.tiff]

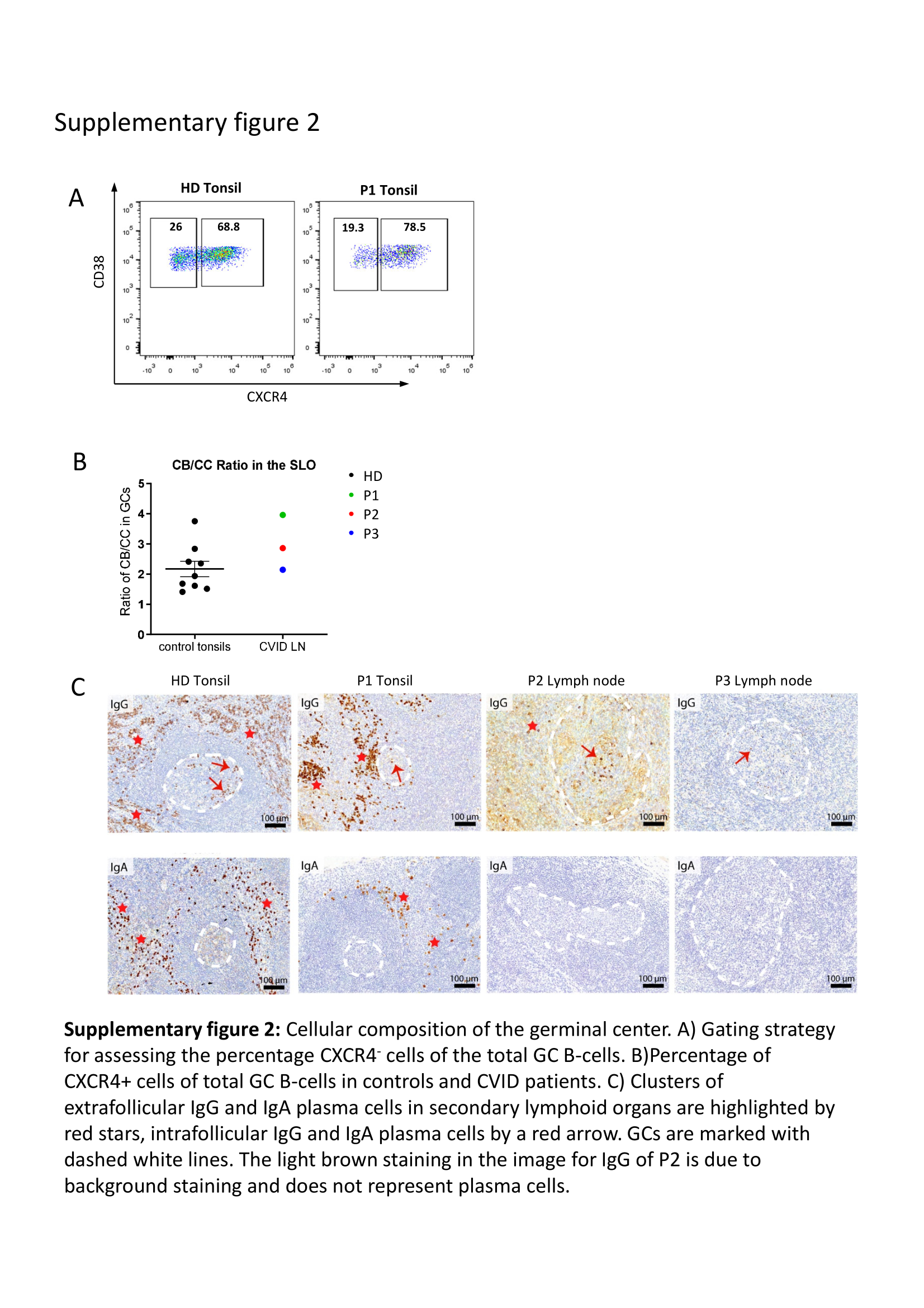

Supplement: Supplementary file 2 [file Image_2.tiff]

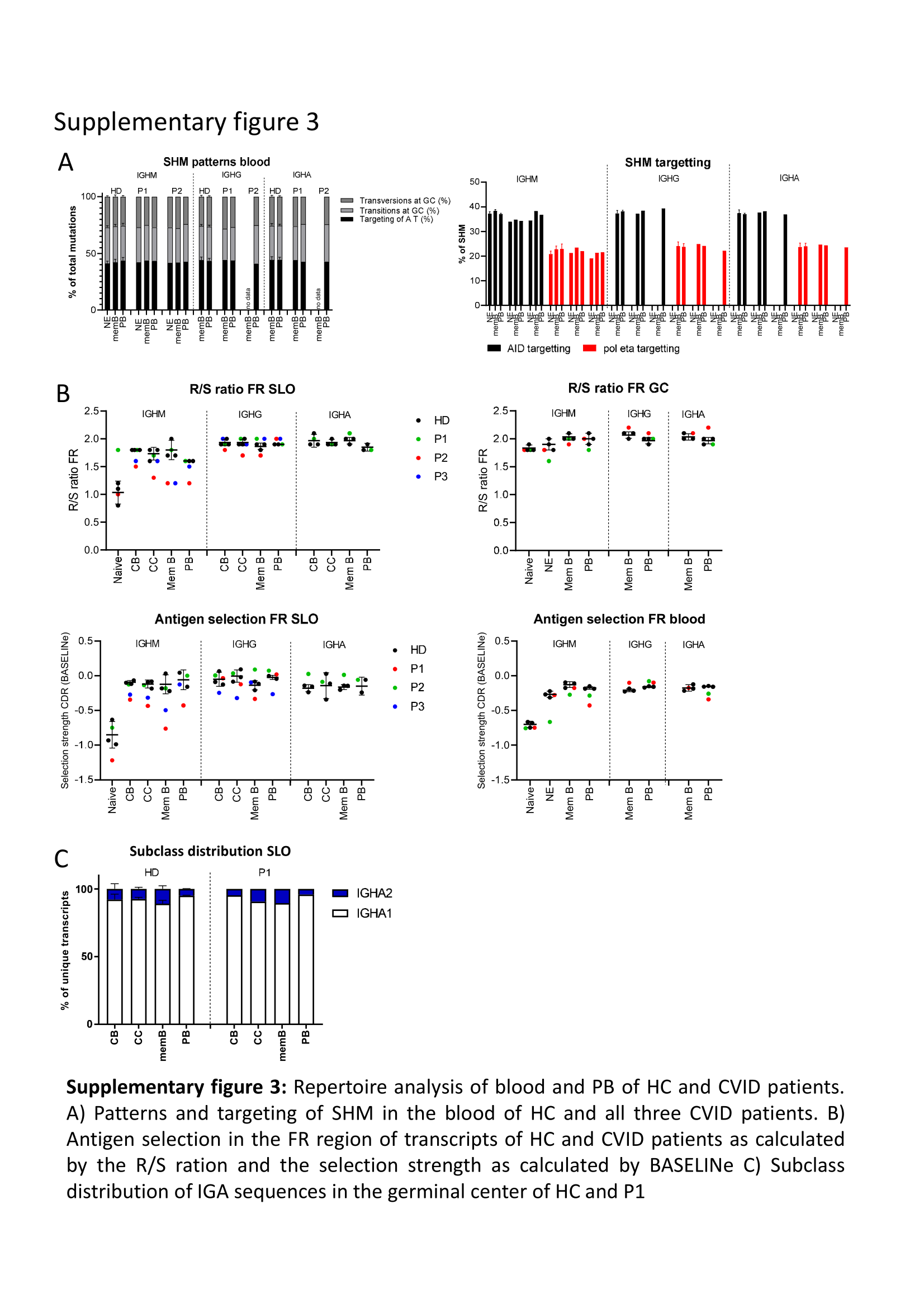

Supplement: Supplementary file 3 [file Image_3.tiff]
