# Supplementary material for: Deciphering imprints of impaired memory B-cell maturation in germinal centers of three patients with common variable immunodeficiency
Source: Front Immunol. 2022 Oct 6;13:959002. doi: 10.3389/fimmu.2022.959002 (PMC9582261; doi:10.3389/fimmu.2022.959002)
Supplement: Supplementary file 4 [file Table_1.docx]

**Supplementary Table 1.** Template specific sequences for BCR repertoire sequencing.

|  | Template specific sequence |
| --- | --- |
| **IGH** |  |
| IGHG Rev | CGATGGGCCCTTGGTG |
| IGHA Rev | GAAGACCTTGGGGCTGGTC |
| Cm CH1 | GGGAATTCTCACAGGAGACGA |
| VH1 FR1 | GGCCTCAGTGAAGGTCTCCTGCAAG |
| VH2 FR1 | GTCTGGTCCTACGCTGGTGAAACCC |
| VH3 FR1 | CTGGGGGGTCCCTGAGACTCTCCTG |
| VH4 FR1 | CTTCGGAGACCCTGTCCCTCACCTG |
| VH5 FR1 | CGGGGAGTCTCTGAAGATCTCCTGT |
| VH6 FR1 | TCGCAGACCCTCTCACTCACCTGTG |
| Jh consensus | CTTACCTGAGGAGACGGTGACC |
