# Supplementary material for: Deciphering imprints of impaired memory B-cell maturation in germinal centers of three patients with common variable immunodeficiency
Source: Front Immunol. 2022 Oct 6;13:959002. doi: 10.3389/fimmu.2022.959002 (PMC9582261; doi:10.3389/fimmu.2022.959002)
Supplement: Supplementary file 6 [file DataSheet_1.docx]

# SUPPLEMENTARY METHODS

## Flowcytometry

Antibodies and kits for flow cytometry included CD3 PerCP (clone SK7), CD4 PerCP (clone SK3), CD11c PE (clone B-ly6), CD21 PE-Cy7 (clone B-ly4), CD27 BV605 (clone L128), CXC5 FITC (clone RF8B2), IFNg FITC (clone B27), IFNg APC (clone B27) (all from BD Biosciences, Franklin Lakes, NJ, USA), CD3 PE-Cy7 (clone UCHT1), CD4 PE-Cy7 (clone RPA-T4), CD45RA FITC (clone ALB11) (all from Beckman Coulter, Brea, CA, USA), CD3 BV605 (clone UCHT1), CD19 APC-Cy7 (clone HIB19), CD19 BV421 (clone HIB19), CD38 PerCPCy5.5 (clone HIT2), CD45 Pacific Blue (clone HI30), CD45RA APC-Cy7 (clone HI100), PD-1 APC (clone EH12.2H7), PD-1 BV421 (clone EH12.2H7), CCR6 BV605 (clone G034E3), IgM BV421 (clone MHM-88), T-bet FITC (clone 4B10) (all from BioLegend, San Diego, CA, USA), CXCR4 PE (clone 12G5), CXCR5 APC (clone 51505), CXCR3 PE (clone WM59) (all from R&D Systems, Minneapolis, MN, USA), IL-17A PE(clone eBio64DEC17) (eBioscience, San Diego, CA, USA), IgD FITC (polyclonal) (Southern Biotec, Birmingham, AL, USA), goat-anti-mouse AF647 from Life Technologies.

## Intracellular cytokine staining

Cryopreserved lymph node mononuclear cells were thawed and resuspended in RPMI medium+10% fetal calf serum (both from Pan-Biotech)+ 100 IU/ml penicillin and 100 μg/ml streptomycin (Invitrogen), rested for 2 h at 37°C and stimulated with 5 ng/ml PMA and 750 ng/ml ionomycin in the presence of Brefeldin A (all Sigma-Aldrich, St. Louis, MO, USA) for 4 h. For intracellular cytokine detection the Cytofix/Cytoperm™ Fixation/Permeabilization Solution Kit (BD Biosciences) was used according to the manufacturer’s instructions.

## Histology

For IgA , CD23 and BCL6 staining, sections were pre-treated with Target Retrieval Solution, pH 9 (Dako, S2367) for 20 min. Slides were incubated with primary antibodies anti-IgA (polyclonal, Dako, IR510) for 20 min, anti-CD23 (DAK-CD23, Dako, IR781) for 30 min or anti-BCL-6 (clone PG-B6p, Dako, IR625) for 2 h. IgA was visualized using the EnVisonFLEX detection kit (Dako, K8000) and for CD23 and BCL6 the EnVision FLEX+ (Dako, K8002) with EnVision FLEX+ Mouse (LINKER) (Dako, K8021) was used.

For IgM staining, sections were pre-treated with Target Retrieval Solution, Citrate pH 6.1 (Dako, S1699) for 30 min. For IgG staining, antigen retrieval was performed using a self-made citrate pH 6.0 buffer. Slides were incubated with anti-IgM (polyclonal, Dako, IS513) and Serum-Free Protein Block (Dako, X0909) for 20 min or anti-IgG (polyclonal, Dako, IR512) for 30 min and visualized with the EnVision FLEX+ detection kit (Dako, K8002) in combination with EnVision FLEX+ Rabbit (LINKER) (Dako, K8019).

## Repertoire sequencing

For sequencing IGH transcript of SLO samples, Roche 454 sequencing was used (1). In short, PCR products were purified by gel extraction (Qiagen) and Agencourt AMPure XP beads (Beckman Coulter). Subsequently, the concentration of the PCR product was measured using the Quant-it Picogreen dsDNA assay (Invitrogen). The purified PCR products were sequenced on the 454 GS junior instrument (Roche) using the Lib-A V2 kit (Roche) according the manufacturer’s recommendations. Data processing was performed using the long-amplicon pipeline 1 from Roche.

## Filtering and removal of sequencing errors from repertoire data

454 sequences were demultiplexed based on their multiplex identifier sequence and 40 nucleotides trimmed from both sides to remove the primer sequence using the ARGalaxy demultiplex tool (2). Illumina FASTQ files were paired (PEAR)(3), demultiplexed, trimmed with cutadapt (4) and converted into FASTA files (5).

FASTA files from both 454 and Illumina data were analyzed using IMGT/High-V-Quest (6). The clonal relation was determined within each class of each population using Change-O with the clonal definition based on the nucleotide hamming distance substitution model with a complete distance of maximal three (7). Clonal assignment was achieved based on the unnormalized first gene segments and the unnormalized distances and for asymmetric distances, the minimal distance was used. SHM, antigen selection and class-switch recombination in ARGalaxy (2) based on one random sequence per clone for the analysis. In populations with only limited read numbers additional filtering to exclude sequencing errors was not feasible. Additionally, due to the location of the reverse primer of the Illumina data, no information on subclass distribution could be obtained from these data. If the above filtering resulted in <45 sequences of one class of one population, this class of this population was excluded from further analysis (**Supplemental Fig. 1A**). Antigen selection was considered impaired if both the R/S ratio and the BASELINe selection strength deviated from the findings in the healthy controls (2).

To assess clonal relation in the GC 454 data, alternative filtering was performed to include all unique sequences of one clone, to exclude sequencing errors (**Supplemental Fig. 1B**). The clonal relation was determined between all sequences obtained from GC populations of a single patient and Homopolymer errors in all sequences containing an insertion or deletion were removed. Subsequently an algorithm based on Ig-Indel identifier was used to distinguish between true SHM and sequencing errors leading to nucleotide substitutions (2, 8). SHM is considered a true mutation if the quality of the base call is above 25 (corresponding to p<0.005) or if the mutation is shared with any other sequences within the same clone. Sequences containing mutations not for filling these criteria were removed from further analysis. Finally, all duplicate sequences (based on the nt sequence and subclass) present in the same population and donor were removed using ARGalaxy to remove any effect of amplification bias (2).

# References

1. IJspeert H, van Schouwenburg PA, van Zessen D, Pico-Knijnenburg I, Driessen GJ, Stubbs AP, et al. Evaluation of the Antigen-Experienced B-Cell Receptor Repertoire in Healthy Children and Adults. Front Immunol. 2016;7:410.

2. IJspeert H, van Schouwenburg PA, van Zessen D, Pico-Knijnenburg I, Stubbs AP, van der Burg M. Antigen Receptor Galaxy: A User-Friendly, Web-Based Tool for Analysis and Visualization of T and B Cell Receptor Repertoire Data. J Immunol. 2017;198(10):4156-65.

3. Zhang J, Kobert K, Flouri T, Stamatakis A. PEAR: a fast and accurate Illumina Paired-End reAd mergeR. Bioinformatics. 2014;30(5):614-20.

4. M. M. Cutadapt removes adapter sequences from high-throughput sequencing reads. EMBnet Journal. 2011;17(1):10-2.

5. Blankenberg D, Gordon A, Von Kuster G, Coraor N, Taylor J, Nekrutenko A, et al. Manipulation of FASTQ data with Galaxy. Bioinformatics. 2010;26(14):1783-5.

6. Alamyar E, Duroux P, Lefranc MP, Giudicelli V. IMGT((R)) tools for the nucleotide analysis of immunoglobulin (IG) and T cell receptor (TR) V-(D)-J repertoires, polymorphisms, and IG mutations: IMGT/V-QUEST and IMGT/HighV-QUEST for NGS. Methods Mol Biol. 2012;882:569-604.

7. Gupta NT, Vander Heiden JA, Uduman M, Gadala-Maria D, Yaari G, Kleinstein SH. Change-O: a toolkit for analyzing large-scale B cell immunoglobulin repertoire sequencing data. Bioinformatics. 2015;31(20):3356-8.

8. Michaeli M, Noga H, Tabibian-Keissar H, Barshack I, Mehr R. Automated cleaning and pre-processing of immunoglobulin gene sequences from high-throughput sequencing. Front Immunol. 2012;3:386.
